# Stereotypical nationality representations in HRI: perspectives from international young adults

**DOI:** 10.3389/frobt.2023.1264614

**Published:** 2023-11-23

**Authors:** Ronald Cumbal, Agnes Axelsson, Shivam Mehta, Olov Engwall

**Affiliations:** Speech, Music and Hearing (TMH), KTH Royal Institute of Technology, Stockholm, Sweden

**Keywords:** accent, appearance, social robot, nationality, stereotype, impression, competence, likeability

## Abstract

People often form immediate expectations about other people, or groups of people, based on visual appearance and characteristics of their voice and speech. These stereotypes, often inaccurate or overgeneralized, may translate to robots that carry human-like qualities. This study aims to explore if nationality-based preconceptions regarding appearance and accents can be found in people’s perception of a virtual and a physical social robot. In an online survey with 80 subjects evaluating different first-language-influenced accents of English and nationality-influenced human-like faces for a virtual robot, we find that accents, in particular, lead to preconceptions on perceived competence and likeability that correspond to previous findings in social science research. In a physical interaction study with 74 participants, we then studied if the perception of competence and likeability is similar after interacting with a robot portraying one of four different nationality representations from the online survey. We find that preconceptions on national stereotypes that appeared in the online survey vanish or are overshadowed by factors related to general interaction quality. We do, however, find some effects of the robot’s stereotypical alignment with the subject group, with Swedish subjects (the majority group in this study) rating the Swedish-accented robot as less competent than the international group, but, on the other hand, recalling more facts from the Swedish robot’s presentation than the international group does. In an extension in which the physical robot was replaced by a virtual robot interacting in the same scenario online, we further found the same results that preconceptions are of less importance after actual interactions, hence demonstrating that the differences in the ratings of the robot between the online survey and the interaction is not due to the interaction medium. We hence conclude that attitudes towards stereotypical national representations in HRI have a weak effect, at least for the user group included in this study (primarily educated young students in an international setting).

## 1 Introduction

As robots are becoming more and more common in different societies across the globe, there is an interest in adapting robots to the local cultural setting, instead of merely assuming that a generic robot design would be suitable everywhere. There is indeed an industry drive to provide possibilities to align a robots’ characteristics with the preference of the majority of local consumers. This trend is particularly evident in the wider support of accents in synthesized voices available on commercial platforms, as offered by Microsoft Azure^1^, Amazon Polly^2^ or Acapela^3^. The trend can also be seen, for example, in the range of rendered human faces offered with the social robot Furhat^4^. However, this approach may in turn trigger stereotypical perceptions by both users that the robot is meant to have been culturally aligned with and users belonging to other cultural groups.

From social science research, we know that interpersonal affinity is shaped by how similar parties in an interaction are to each other. Similarity-Attraction Theory suggests that people are more attracted to others who share qualities like attitudes, personalities and physical attributes with them (Byrne et al., 1967; Singh and Ho, 2000). This phenomenon may further extend to the way people treat social non-human agents. Nass et al. (1994), for example, argued that humans apply human-human social rules to their interactions with computers. More recently, Gambino et al. (2020) presented the idea that humans instead create special human-media social rules to apply to their interactions with artificial social agents and computers. In both of these cases, social dynamics that originate in interactions between humans are applied to, or extended to, interaction with non-human systems.

When humans apply social rules to an interaction with an embodied agent, stereotypes based on the features of the agent can follow with the application of those social rules. Kurylo (2012) presents a thorough literature study on the term *stereotype*, and shows that non-scientists generally think of stereotypes as a way to form expectations of someone that they have not seen before, i.e., a functional view. Since people interacting with social robots and similar agents are often uncertain of the capabilities and capacities of the agents with which they are interacting (Spence et al., 2014; Kwon et al., 2016), it is likely that their impressions of the agent will be informed by their own social beliefs. It is then hard to predict how the stereotypes produced by features of an agent (voice and/or appearance) may apply to social robots and similar agents.

Since social robots with human-like characteristics become more frequent in research and commercial settings, many of them carrying clear cultural connotations like the robots Sophia^5^, Ameca^6^ or Erica^7^, it is important to investigate if and how these characteristics induce social stereotypes. In this study we explore how different nationality-encoded representations, both in the accent and facial appearance of a virtual and physical social robot, affect the perceptual and functional dimensions of people interacting with the robot. To this aim, we first use an online survey with a comprehensive set of appearance- and accent-based nationality representations to measure the immediate perception of competence, likeability and human-likeness, following studies on first impressions in human-human exposures, such as the one by Willis and Todorov (2006). In a separate study, four selected nationality-representations with differing voice and appearance attributes are then evaluated on the same dimensions after an interaction with a physical social robot, in order to investigate both differences between nationality-encoded robots and to compare perception after interaction to that of first impressions. The latter is of interest since psychological studies on human-human interaction (Gregg et al., 2006; Mann and Ferguson, 2015) have shown that perception after interaction is similar to first impressions and it is hence valuable to investigate if this also holds for human-robot interaction. We also explore the behavioral dimension of interacting with nationality-encoded robots by measuring how much participants recall from the interaction. It is understood that when speech diverges from a listener’s expectation, additional cognitive processes are required to aid in comprehension. Even when accented speech is intelligible to listeners (i.e., they can accurately reproduce or transcribe it), there is more cognitive demand compared to processing native accents (Van Engen and Peelle, 2014). In alignment with these findings, we emphasize the significance of examining accented speech in current and future development of human-inspired social robots. Furthermore, we conduct an extension study, in which two of the nationality representations are investigated in an online setting with a virtual version of the robot, in order to investigate if the difference between situated and mediated interaction has any effects on the perception of the robot. We report on the results of the three studies and discuss the relevance of these findings to current and future development of human-inspired social robots.

## 2 Ethnicity, nationality and culture

Before we can turn the attention to stereotypes related to robots’ appearance and accents, it is important to specify the terminology used, since many diverse definitions have been proposed. The terms *ethnicity* or *nationality* are commonly used to define an individual’s cultural background or descent. While these terms can be interpreted in different ways and do not share a common definition, a clear distinction between them is often hard to establish (Conversi, 2003). In the real world, these are often synonyms to denote an origin, that, together with skin colour or descent, mark attributes protected by international law against possible discriminatory actions (Banton, 2004). This approach to mark similarities among nationality and ethnicity, based on ancestors’ countries of origin, however, may also be subjective to the individual choice of self-identifying with that origin. Hence, as Baton notes, “in this domain, ethnic and national origin do go together, but they have to be considered along with other bases of group formation…and other grounds of prohibited discrimination…as listed in the Universal Declaration of Human Rights” (Banton, 2004).

With the same objective purpose, the terms *nationality* and *ethnicity* are commonly interchanged in scientific studies that address bias or stereotypes in vision-based technology, even though the relation to an individual’s origin is often lost in the process. For example, when evaluating the composition of datasets, Hirota et al. (2022) alternate between ethnicity-race (e.g., Asian, Caucasian, Black) and ethnicity-nationality (e.g., American, Chinese, Indian) to study race bias. The National Institute for Standards and Technology (NIST) uses nationality as a proxy for ethnicity to study its relation to gender classification (Ngan and Grother, 2015), and Farinella and Dugelay (2012) apply a binary classification (Caucasian and non-Caucasian) to investigate gender and ethnicity image classification. Recent efforts to consolidate objective guidelines when handling bias or stereotypes in (primarily) image datasets have acknowledged the inconsistency of the categories ethnicity and nationality, but have highlighted the large variability of a subject’s phenotypic features within ethnic categories as an important root for this ambiguity (Buolamwini, 2017). Buolamwini instead propose to use skin type as a more visually precise label to measure diversity in a dataset. This solution, while optimal for the examination of bias in image datasets, removes the notion of a subject’s origin as an additional indicator of source for stereotypes. When evaluating stereotypes in speech-based technology, the dimensions of ethnicity or nationality take the form of accents, or dialects, to differentiate social groups and the individuals who compose them. As such, these terms are also used interchangeably when addressing the mapping of an accent to an individual’s identity or cultural background. A common classification in these studies is the division of native and non-native speakers/speech, although this terminology may itself introduce negative preconceptions Cheng et al. (2021).

Definitions of the term *culture* have an even greater diversity Baldwin et al. (2006) and may relate to, e.g., societal, professional, gender and age groups as well as to nationality. Cultural belonging may influence perceptions of stereotypes in many different ways, going beyond facial appearance and accent, which are the focus in this work. Stereotypes may, e.g., be formed based on gender and match of gender in an interaction, and, as discussed in Section 5.1, we therefore only include synthetic voices of one gender (male) to reduce influence of this cultural factor. Stereotypes may further be formed based on societal hierarchy and age (i.e., if the interlocutor is perceived to have a higher or lower status than the observer due to, e.g., assumed experience, social class or profession). We reduce such effects by selecting a participant pool that is relatively homogeneous when it comes to age (younger adults) and educational level (mainly university students). While we acknowledge that this may affect the generalisability of the results—they will hold for the given context and users of a certain age group and educational background, but not necessarily for other contexts and users—it focuses the study on stereotypical perception linked primarily to nationality, as influence from other cultural factors is reduced.

Thus, in this article, we consider cultural belonging to specifically be constituted of the context in which the individual exists. Factors playing into this include present country of residence, and an individual’s nationality. To explore this, we will focus on one societal context, i.e., Sweden, and individuals who are either nationals or are of a different nationality in this context. As all participants share the societal context of being residents of Sweden, we will categorise them by nationality, rather than culture. We further employ the term *nationality* to categorize the stereotypical cultural identities that are represented in the virtual or physical social robots. Since accents in a *lingua franca* are often segmented by national boundaries, and, in comparison to appearance, voice may be a stronger proxy to determine people’s perception of an individual’s cultural categorization (Rakić et al., 2011), it is reasonable to use this terminology throughout the paper. We acknowledge, however, that any classification of identity may itself carry problematic connotations and may not conform to different individual’s self-identification (Jo and Gebru, 2020). Furthermore, it is specially important to clarify that in this work we do not promote specific robot designs that should imply a cultural identity, instead we evaluate those presentations that are already available in commercial and research platforms.

## 3 Related work

### 3.1 Stereotypes

The traits given to a robot are likely to influence its users’ perception of and interaction with it, just as previous findings from social science research suggest that nationality traits are associated with positively and negatively coded stereotypes and that (mis)alignment with the majority population affect these. For example, Phalet and Poppe (1997) evaluated stereotypes in the European context, finding that, with respect to morality and competence, participants showed more positive views towards Western than Eastern European countries. Mlicki and Ellemers (1996) showed that students of both Dutch and Polish nationality tended to assign negative stereotypical traits to Polish nationality, while assigning positive values to Dutch nationality. Regarding accent, Lindemann (2005) found that native English speakers in the United States had negative views of accents attached to all second language speakers, except towards (Western) Europeans. The participants assigned negative characteristics to “Chinese” and “Mexican” English, and described Russian accent as “harsh” and “guttural”, while the views on Indian and German English varied.

Thus, if a social agent is given an accent and/or appearance matching that of its users, then the users may get a feeling that they and the robot belong to the same social group (Torre and Maguer, 2020). This can come with either positive (Dahlbäck et al., 2007) or negative (Niculescu et al., 2008; Torre and Maguer, 2020; Batsaikhan et al., 2021) impressions depending on what the user thinks about their own social group’s competence and standing in the task being performed by the agent—or how well the agent portrays the social group (see the comment in Section 3.2 regarding the study by Haring et al., 2014). Additionally, whether the user is reacting to a local accent of their native language (Torre and Maguer, 2020), a second language (Dahlbäck et al., 2007; Niculescu et al., 2008) or a local dialect of a *lingua franca* (Batsaikhan et al., 2021) appears to interact with the other effects.

If, on the other hand, a social agent is given an accent or appearance mismatching its users, negative stereotypes may be applied to the social agent based on the user’s perception of the group the robot represents. In an experiment by Eyssel and Kuchenbrandt (2012), German participants interacted with a robot that only differed in whether it was described as being developed by a Turkish or German university, with, respectively, an accompanying Turkish-coded or German-coded name. Participants rated the German-coded robot as having a higher degree of warmness and psychological closeness than the Turkish-coded robot. This indicates that out-group effects can be found even when the group classification of the system is only implied and not based on any physical characteristics. Spatola et al. (2019) suggested that the effect gets stronger the more human-like a robot is.

### 3.2 Appearance

One aspect of a human or human-like social agent that informs users’ stereotypes is appearance. McGinn and Torre (2019) showed that experiment participants linked specific voices to specific robot appearances in ways that were consistent across participants, but were not connected to either the actual voices used by the robots pictured, or based on the linguistic content spoken by the voices. In a study exploring the perspective of “race”^8^ in robotics, Bartneck et al. (2018) used the *shooter bias paradigm* experiment to evaluate if participants would react in the same way to racially designed robots (“Black” and “White”)^9^ as to humans of different skin colour. The results confirmed that people attributed a race to the robots and had equal reactions to the robot racialized as Black as to a stimuli portraying a Black person. In a subsequent essay, Sparrow (2020) argued that humanoid social robots are likely to be associated with different races, most frequently with “White” people, but debated that efforts to shift this representation to other races might produce worse outcomes, e.g., associating the servant role of a robot to other races. Sparrow (2020) proposed an alternative where robots are designed with colors that cannot be assigned to a race, e.g., blue or green, or suggested to design robots with less humanoid characteristics, although acknowledged that the latter may be in conflict with the tendency to create more “social” robots by making them humanoid.

In terms of nationality representation, in a study by Haring et al. (2014), Australian and Japanese subjects evaluated a social robot that was designed to look like a Japanese woman, on multiple metrics before and after interacting with it. After the interaction, Australian participants rated the robot more highly on anthropomorphism, likeability and perceived intelligence than the Japanese participants. Additionally, the Australian participants trusted the robot more after playing an economic game together. While Haring et al. take this result to mean that Japanese participants are more used to and, therefore, more skeptical to the abilities of social robots, another interpretation that we give is that the robot was more convincing as a member of the Japanese social group to those who were not part of it than to those who were. Makatchev et al. (2013) further explored the effect of facial appearance matching verbal and non-verbal behaviours when representing ethnicity^10^ in social robots. In this study, participants were exposed to the robot Hala, composed of a head displayed on a screen and a robotic torso. The robot was designed with face and behaviour cues of native speakers of American English and native speakers of Arabic speaking English as a second language and was assessed by the corresponding subject groups. The results showed that the selected behaviours generated a stronger signal of ethnic attributes with the “American” robot rather than the “Arabic” robot. The contribution of the robot faces was less relevant, but showed potential complex interactions with the selected behaviours. Furthermore, there was no evidence of the effect of *homophily* after the interactions, i.e., the tendency of individuals to associate disproportionately with others who are similar to themselves.

These studies demonstrate that people indeed are influenced by the appearance of a robot. When this appearance resembles attributes associated with nationality (Section 2 for a discussion on these terms in relation to this study), people are able to perceive this representation, and more importantly, behave as if these characteristics were part of the identity of the robot. Notably, other features, like gestures and voice, can influence this perception.

### 3.3 Accent

In human-human interactions, a listener’s perception of a speaker’s accent affects which stereotypes the listener applies to the speaker, regarding, e.g., the speaker’s background (McGinn and Torre, 2019). For example, when examining the accent attitudes across the United Kingdom, Sharma et al. (2022) found that British accents associated with speakers of a working class in urban and rural areas were rated as “less prestigious” and “less pleasant” than accents linked to higher social status, e.g., royalty. Notably by replicating studies from 1970 to 2005, the authors found that these rankings were shown to have varied very little over a long period of time, with only a slight improvement in lower rankings. Differences in how accents are perceived are further especially clear when asking experiment participants to rate their perception of a *lingua franca*. Ladegaard (1998) provide an example of this, showing that Danish experiment participants rated British speech as more cultured and American speech as more exciting, with Australian speech being rated as more rural and approachable, even by participants who could not identify the speech they were listening to as specifically Australian. For Chinese, Batsaikhan et al. (2021) showed that Singaporean participants rated Standard Chinese as more trustworthy than Singaporean-accented Chinese. Similarly, Obremski et al. (2022) found that German subjects were more negative towards a robot with German-accented English than were native English speakers and that the opposite was true for a native English-speaking robot. Moreover, these perceptions may be altered with extended exposure to diversity. For example, Boduch-Grabka and Lev-Ari (2021) recently presented work indicating that time spent studying abroad and exposure to non-native accents of English may increase acceptance of accented speech.

Research on accented voices used with (dis)embodied agents has further shown similarities to the above described social phenomena. In an experiment by Dahlbäck et al. (2007), 96 Swedish and American participants interacted with voice agents reading out tourist facts about a Swedish and American destination. Participants preferred a voice with an accent matching their own when reading out tourist information about both the Swedish and American destinations. Although the results appear to contradict those on Singaporean Chinese by Batsaikhan et al. (2021) and German-accented English by Obremski et al. (2022), the comparison is complicated by the differing scenarios. A more direct comparison can be found in work by Niculescu et al. (2008), who compared Singaporean English speakers’ impressions of a Singaporean English-accented virtual help desk assistant to one with a British English accent. The British English-accented agent was rated as significantly more polite than the Singaporean English voice. Niculescu et al. argue that this is because of cultural effects and attempts by the Singaporean government to minimise use of Singaporean-accented English. These factors would not apply to the Swedish-American comparison of Dahlbäck et al. (2007). Another study exploring local and global accents in English was performed by Andrist et al. (2015). In this work the authors set up an experiment where a local variety of English and Arabic were compared to a standard English and Arabic variety, respectively. Participants in the experiment were asked to rank suggestions from two robots in a travel guide task by how likely they were to follow one suggestion or the other. In both English and Arabic, robots that displayed less knowledge and had less structured rhetoric were less likely to have their suggestions followed. For Arabic, the local dialect was unlikely to have its advice followed when in the low knowledge, low rhetoric mode, while instead being significantly likelier to have its advice followed in the high-knowledge, high-rhetoric mode when compared with the standard Arabic variety. This indicates that participants valued rhetoric and display of knowledge more in the local dialect than in the standard language. In an experiment run by Torre and Maguer (2020), 499 British English speakers from a variety of regions of the UK preferred a robotic agent to have a Standard Southern British English (SSBE) accent, with a Liverpool accent and the responder’s own regional accent ranking the lowest. Foster and Stuart-Smith (2023) recently presented a preliminary study evaluating users’ expectations for conversational interactions with a robot. The authors focused on evaluating social factors when rating different Scottish English accent and dialects among residents of Glasgow. The results revealed that participants expected to better understand a—standard-English—speaking robot, with middle-class and male respondents providing more positive answers, supporting the trends shown by Sharma et al. (2022). Although these studies appear to contradict the results of Dahlbäck et al. (2007), it is not obvious how one should compare first-language speakers’ impression of their own dialect to second-language speakers’ impression of their own accent.

These studies show that people assign different values to speech with different accents or dialects and, although these perceptions vary across populations, there is a slight preference towards “standard” forms of spoken languages, which is also replicated with synthesized speech used in (dis)embodied agents. While these patterns appear to remain over longer periods of time Sharma et al. (2022), it is important to note that these perceptions may be altered with extended exposure to diverse cultures Boduch-Grabka and Lev-Ari (2021). Given the recent efforts to expand the options of accented English voices in commercially available Text-to-Speech (TTS) systems—which are often used with virtual or robotic agents—it is important to extend the knowledge of how globalized populations, e.g., international university students with greater exposure to different cultures, perceive these culturally diverse voices when used by virtual or robotic agents.

## 4 Research objectives

This work presents a study to explore user perceptions on representations of nationality—based on accent and appearance—of a virtual and physical social robot; and how perception of these representations may contain social stereotypes. These objectives are formalized with the following research questions:

• How do individuals form perceptions of a social virtual robot when exposed to *brief stimuli*, considering the robot’s accent and appearance indicate a certain nationality?
• How do individuals perceive a social physical robot whose accent and appearance indicate a certain nationality, following a short interaction?
• Extension: How do individuals perceive a social virtual robot whose accent and appearance indicate a certain nationality, following a short interaction?
• Secondary aim: Are there any differences in how individuals of the nationality portrayed by the [physical or virtual] robot perceive it, compared to an international group, and compared to a robot that is portraying another nationality?

These objectives hence also explore, in addition to the effects of robot nationality, differences between brief stimuli and short interactions, between virtual and physical settings. We further address the question of if aligning the robot’s non-native accent of a lingua franca to a sub-group influences their perception of the robot.

## 5 Nationality representations

In this work we manipulated the robot’s vocal and facial characteristics to suggest different representations of nationality. We acknowledge that assigning simple facial and voice characteristics to a nationality—incorrectly—reduces this intricate social construct to two dimensions. However, it is known that people use such individual factors to presume interlocutor’s background and build mental models from them, which further translates to (dis)embodied agents (Nass and Brave, 2005; Spatola et al., 2019).

### 5.1 Appearance and accent

**FIGURE 1:**
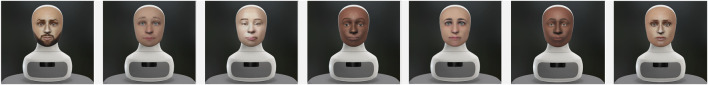
A selection of seven nationality-influenced faces available as part of the robot Furhat FaceCore package.

We employed the robot Furhat, in its virtual and physical form, to display different representations of nationality. For appearance, we used the rendered faces with a realistic skin texture from the upgraded *FaceCore* package, as shown in Figure 1. We refrained from rendering additional faces using the available toolkit to avoid introducing confounding variables or personal biases in new designs. Although we recognize the limited range of this distribution, the chosen selection serves as a clear representation of commercially available robot face designs that embody national identities. As a result, these representations are likely to be spread among the public and may induce social stereotypes. These faces have no advertised gender attribute.

To create accented speech in English, we employed three different Text-to-Speech (TTS) options. We first fine-tuned the OverFlow TTS model with recordings from different speakers present in the L2-arctic corpus (Zhao et al., 2018). OverFlow TTS is an extension of neural HMM TTS that uses Hidden Markov Models (HMMs) to carry the voice characteristics of accented speech from a first language (L1) to an L2-English speaker in a low-resource setting (Mehta et al., 2023). We secondly used officially distributed accented English voices from Google TTS^11^ and Microsoft Azure TTS^12^ (e.g., Singaporean, Nigerian, British and American English). Thirdly, we used Google TTS to process English text with different language models to simulate additional accents. This results in exaggerated, but still plausible, accented speech, since the English text is being synthesised as being read by a non-native speaker applying pronunciation rules from the own language (e.g., Franglais, Denglish or Swenglish). The result is a heavily accented English that is nevertheless representative of a subgroup of non-native speakers from the applied language model, as long as the text does not contain numbers or words from the first language (as these will be produced as in the first language rather than as accented English).

In this process, we made the decision to use only voices assigned to the male gender from the off-the-shelf TTS services and data from speakers reported as male in the L2-arctic corpus. This decision reduces the factors to consider in our analysis, since gender perception in voice synthesis undeniably carries additional components of stereotypical nature. In particular Loideain and Adams (2020) argued that the choice of female voices for voice assistants reinforces the stereotype of women being submissive entities without agency. We recognize that gender is a strong factor in attitudes toward (dis)embodied agents and guide readers to the work of Danielescu et al. (2023) in Non-Binary TTS research, as well as the comprehensive review of gender in humanoid robots by Perugia and Lisy (2022).

## 6 Interaction context

Throughout the following studies, both the virtual and physical social robots were introduced to participants as an art presenter for a museum exhibition. Museum exhibitions typically draw individuals from various socio-demographic and ethnic backgrounds, as well as different age groups and educational statuses (Vom Lehn et al., 2005). These diverse characteristics serve to stimulate the development and further exploration of intricate social interactions between human beings and robotic platforms. Previous research on the effect of accents has further found that different contexts elicit different responses from people interacting with agents (Salem et al., 2013; Andrist et al., 2015). Therefore, this role is selected to convey an image of knowledge and competence for the robot, with the aim that this should evoke stronger attitudes toward the virtual and physical social robot in complex social interactions. Furthermore, we focused on the English language to reflect its predominance in international environments, i.e., as a *lingua franca*, as well as the relevance and domination of English voices in the market of digital personal assistants and speech interfaces (Moore, 2017; Aylett et al., 2019), usually employing “standard” accents for these devices (Foster and Stuart-Smith, 2023).

## 7 Online survey

The online survey had the objectives of 1) validating the accent and appearance of a virtual robot to a perceived representation of nationality, 2) selecting a subset of facial appearances and accents suitable for a comparison of different robot nationality encodings and 3) collecting ratings on the virtual robot corresponding to first impression perception.

### 7.1 Method

We employed the digital platform cognition.run to develop the online study. Participants began by reporting age, nationality, gender, first language, English proficiency (fluent or not), and experience with voice assistants, virtual agents and speaking interactive robots (*None*: 1—*Expert*: 5). Participants were then requested to individually evaluate a set of accented voices that reproduced the first two sentences of the Rainbow Passage^13^. This text is often used in speech synthesis evaluation as it contains most sounds, or combination of sounds, found in the English language. On average, each audio segment had an 11 s duration. Through this web interface, the participants assigned a gender and nationality to the voice, rated how easy or difficult it was to understand the utterance (*Very difficult*: 1—*Very easy*: 5), and were asked to rate the perceived level of naturalness (*Not natural at all*: 1—*Completely human-like*: 5), perceived competence (*Very Low*: 1—*Very High*: 5), perceived English proficiency (*Beginner*: 1—*Native*: 5), and likeability of the voice (*Not at all*: 1—*A Great Deal*: 5). The list of countries presented to the participants when selecting a nationality corresponded to the TTS options described Table 1. In the final section, participants were asked corresponding assessment questions regarding nationality, gender, competence, naturalness and likeability when presented with a picture of the 7 individual virtual robot faces shown in Figure 1. The stimuli order was randomized for each participant.

**TABLE 1:** Results from online surveys evaluating different accented voices on perceived Nationality, easiness of Understanding, Naturalness, perceived Competence, Likeability, and perceived English Proficiency. Subscripts *O* indicates voices trained with the OverFlow technique, subscripts *Go* and *Gs* those produced with Google TTS (respectively, official and simulated accents), and subscript *M* those with Microsoft Azure TTS. All voices were perceived as belonging to a male speaker. Numbers in brackets indicate exclusion criteria listed in Section 7.2. → indicate voices selected for the robot interaction study.

L1/origin accent	Nationality (%)	Understand	Natural	Competent	Likeable	Proficient
*Arabic*_*O*_	Iran (29), PL (19)	2.60 [1]	2.87	2.13	2.04	1.73
→ *British*_*O*_	UK (81), US (6)	3.25	2.26	3.19	2.50	3.77
*Mandarin*_*O*_	NL (15), CN (10)	3.06	2.47	2.63	2.08	2.65
*Nigerian*_*O*_	Kenya (31), NG (27)	2.90 [1]	2.89	2.73	2.40	2.33
*USA*_*O*_	US (83), NL (6)	3.50	2.55	3.19	2.40	3.96
*British*_*Go*_	UK (92), US (8)	4.83 [2]	3.77	4.38	3.88	4.75
*Hindi*_*Go*_	India (63), SY (4)	3.92	3.45	3.42	2.98	3.40
*USA*_*Go*_	US (88), UK (10)	4.60 [2]	3.47	4.04	3.25	4.38
*Arabic*_*Gs*_	Iran (25), SY (23)	2.21 [1]	2.19	2.33	1.90	2.08
*Dutch*_*Gs*_	NL (52), US (6)	3.52	2.49	2.98	2.50	2.98
*French*_*Gs*_	France (81), NG (8)	3.02	2.36	2.67	1.96	2.44
*German*_*Gs*_	DE (58), FR (33)	3.58	3.19	3.29	2.69	2.96
*Japanese*_*Gs*_	Japan (77), CN (6)	1.38 [1]	2.43	2.04	2.21	1.35
→ *Mandarin*_*Gs*_	China (29), DE (17)	3.08	1.94	2.77	1.90	2.54
*Portuguese*_*Gs*_	Brazil (38), PL (29)	2.27 [1]	2.60	2.33	2.13	1.96
→ *Swedish*_*Gs*_	Sweden (75), KR (6)	3.25	2.32	2.83	2.29	2.65
→ *Kenyan*_*M*_	Nigeria (46), KE (25)	3.81	3.55	3.44	3.25	3.33
*Nigerian*_*M*_	Nigeria (50), KE (21)	3.92	3.62	3.40	3.23	3.21
*Singaporean*_*M*_	SG (19), ZA (15)	3.73	2.83	3.08	2.79	3.04
*SouthAfrican*_*M*_	Kenya (25), ZA (19)	4.02 [2]	3.47	3.40	3.15	3.25

Two versions of the same online survey were published, in October 2022 and April 2023. The first survey consisted of 5 OverFlow voices, 3 commercial accented Google TTS voices and 8 simulated accented Google TTS voices using transferred language models. The second version included only 4 trained Microsoft Azure voices (and no face appearance evaluation). The two sets were assessed by two different rater groups and are shown at the top and bottom of Table 1, respectively. These online surveys were advertised using the prolific.co platform. The rate of compensation was defined at 15–20 GBP per hour (approx. 18–25 USD). Participants were pre-screened for (self-reported) Swedish residence and English language proficiency.

### 7.2 Selecting representations of nationality

The results of the online survey were used to define four perceptually validated representations of nationality that were to be evaluated in the (embodied) robot study. The following exclusion criteria were used regarding the survey results:

1. Remove voices that are significantly lower than the average level of understanding, to avoid voices that would be too difficult to understand when interacting with the robot.
2. Remove voices that are significantly higher than the average level of understanding, to avoid the risk of biasing the experiment towards a test of TTS quality rather than of accent.
3. Remove voices or faces that have a clear mismatch between the intended and perceived representation of nationality.

This process was designed to guarantee that the options evaluated in the robot study had comparable levels of naturalness (as well as easiness of understanding for voices). In addition, the online survey further aimed at identifying four national representations that firstly had strong differences in perceived competence and likeability and secondly had a reasonable geographic spread (i.e., representing one native accent, one European accent aligned with the majority subject group, and one Asian and one African accent).

## 8 Physical robot study

The study was designed to assess how the results obtained in the online survey translated to an interaction with a physical social robot. Although, Section 5 acknowledges that countries outside of Western Europe and the Global North may generally receive less positive judgments on different perceptual factors, there is a diverse set of results when evaluating these stereotypes in technology-based interactions. As described in Section 3, the perceptions of culturally encoded appearances and voices can vary depending on if the accent is native or non-native, if the agents’ nationality-representation match that of the participants’ (Dahlbäck et al., 2007; Niculescu et al., 2008; Torre and Maguer, 2020), their prior experience with such technological interactions (Haring et al., 2014), and the overarching stereotypes associated with different cultural groups (Ladegaard, 1998; Batsaikhan et al., 2021). Therefore, to construct our hypotheses, we use these previous studies and the results gathered in the online survey to evaluate if the first impressions of the nationality-encoded virtual robots hold for a short interaction with a physical robot. The hypotheses are formalized as follows:

• **H1:** Participants will give higher ratings for *perceived competence* and *likeability* to the robot that scored higher values in these dimensions in the online survey.
• **H2:** Participants will have better *recall* of information provided by the robot that scored higher values in *perceived competence* and *likeability* in the online survey.
• **H3:** Participants will give higher ratings to the robot and have better recall of information, if the robot has a nationality-representation *aligned* with the participant’s own nationality.

It should be noted that these hypotheses are formulated in order to formalise the study, rather than signalling expectations regarding the results, since previous work have indicated, e.g., that users’ perception of an agent may change between first impression and a longer interaction (Paetzel and Castellano, 2019; Paetzel et al., 2020) and that subjects may—or may not—be more positive towards an agent that is portrayed as having their own nationality (see Section 3).

### 8.1 Method

#### 8.1.1 Interactive presentation

**FIGURE 2:**
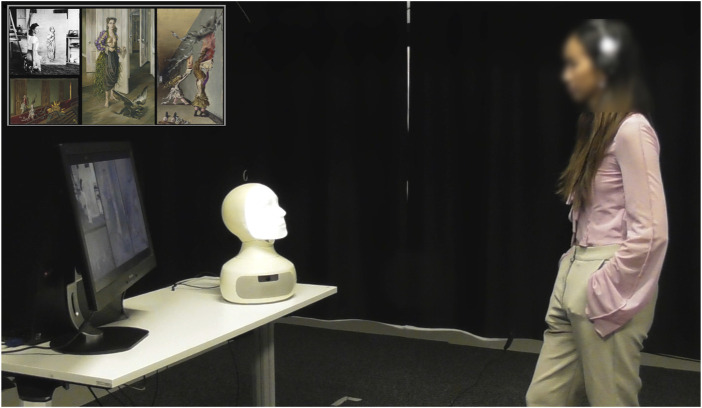
A participant interacting with the robot Furhat as part of the physical robot study. To the left of the robot Furhat, a monitor shows images relating to the presentation (expanded in the top left corner of the figure).

A dialogue system created by Axelsson and Skantze (2023) was used to allow the robot to present an artist’s biography and artwork to each participant. The presentation system selected statements in real-time by querying a custom-made knowledge graph containing information from the artist’s Wikipedia entry. The selected properties were lexicalised using GPT-3. In this study a static mode of the dialogue system was used, i.e., it did not adapt to the reactions of the users, unlike the version presented in (Axelsson and Skantze, 2023). To compensate for the lack of adaptation, an additional question-answering module was implemented using GPT-3. When users asked a question, GPT-3 was queried to determine what the robot should say based on the entire dialogue history. This gives the system some answering capability by repeating or rephrasing previously presented information. To reduce the possibility that participants had previously encountered any information exposed in the presentation, a relatively less know artist was chosen: Dorothea Tanning, an American painter, sculptor, writer, and poet, known for her work influenced by Surrealism^14^. As shown in Figure 2, the presentation was aided by a collage of photos and paintings of the artist displayed on a large computer monitor. During the presentation, the robot glanced and referenced to these items when appropriate.

#### 8.1.2 Measures

Following previous research on stereotypes and interactive agents, we measured aspects related to social constructs, i.e., competence (Krenn et al., 2017), and factors used to evaluate interactive robots, i.e., likeability, to evaluate the effects of the nationality representations. From the Partner Modelling Questionnaire (PMQ) (Doyle et al., 2021) we use the dimensions of *Partner Competence* *&* *Dependability* and *Human-likeness*, and from the Godspeed questionnaire (Bartneck et al., 2009) we use the sections for *Likeability* and *Anthropomorphism*. Although the dimensions of *Human-likeness* and *Anthropomorphism* may be related, in this study we use the former (from the PMQ) to evaluate the interactive (speaking) characteristics of the robot, while the latter (from Godspeed) focuses on concepts connected to the human shape or form. The final questionnaire, implemented in Google Forms, also included questions used in the online survey, i.e., selecting gender and nationality (including a *“None”* option, unlike in the online survey) for the robot, report on the participant’s experience of speaking with interactive robots (*None*: 1—*Expert*: 5), rate how easy it was to understand the presentation (*Very difficult*: 1—*Very easy*: 5), and report the perceived English proficiency of the robot (*Beginner*: 1—*Native*: 5). The measurements of *anthropomorphism* and *human-likeness* were used to verify that these aspects were equal between robot conditions, and that our manipulation influenced only *perceived competence* and *likeability* differences.

A quiz was used to evaluate how much information participants recalled after the presentation and consisted of seven multiple-choice questions formulated from the information used to develop the custom-made knowledge graph. For each question, the quiz choices consisted of one correct answer, three distractors, a *“None of the options”* alternative and a *“This information was not presented”* option. Two of the seven questions served as distractor questions that were not possible to answer based on the information presented by the robot, but *“none of the above”* and *“not presented”* were not counted for these questions, even if it was correct. The order of the response-choices was randomized for each participant, but not the order of the questions.

#### 8.1.3 Procedure

The study started by the participants signing the consent form, which included the instructions for interacting with the robot, the right to leave participation voluntarily, the right to refuse to answer questions, and the right to prohibit the use of media obtained from the study in any scientific publications. The participants were then welcomed inside the lab where the robot was situated, as shown in Figure 2, and, once any doubts regarding the experiment had been cleared, participants were informed that the robot would begin the presentation soon. The experimenter then initiated the autonomous presentation. When the system had run out of new statements in the custom knowledge graph, the robot finished the presentation, said goodbye and shut down. The participants were then asked to complete the quiz about the presentation and fill out the post-questionnaire. The experiment leader debriefed the participants on the purpose of the study and the autonomy of the robot. Finally the participants received a compensation of 100 SEK (approx. 9.58 USD).

## 9 Results

### 9.1 Online survey

In total, 20 synthesized voices representing 15 English accents were evaluated in the online surveys. Those included in the first version are displayed at the top of Table 1, with the subscript *O* for those trained with the OverFlow technique, and the subscript *Go* and *Gs* for those produced with the Google TTS service as official or simulated accents, respectively. The results shown at the bottom of Table 1 correspond to the second online survey with the four voices from Microsoft Azure, identified with the subscript *M*.

The initial instance of the survey had 35 participants, which included 3 valid pilot tests, while the second survey had 36. In a effort to balance the distribution of participants’ nationalities, 18 more individuals were recruited. The data from 9 participants were removed for not completing all attention checks. Out of the final 80 participants, 31 reported themselves as female, 2 as non-binary and 47 as male. The average age of the participants was 31.2 years (SD: 9.71). All participants were residents of Sweden, 44 of Swedish nationality and the rest corresponding to Portugal (4), India (4), Canada (2), Finland (2), Poland (2), Azerbaijan, Brazil, Colombia, Czechia, Estonia, Germany, Greece, Indonesia, Ireland, Israel, Italy, Kenya, Korea, Netherlands, Latvia, Russia, Spain, Syria, Turkey, Venezuela, United Kingdom and United States. When non-Swedish participants’ nationalities are grouped using the framework of the United Nations Regional Groups^15^, 47.2% of those participants correspond to the Western European and Others Group (WEOG), 22.2% to the Eastern European Group, 19.4% to the Asia and the Pacific Group, 8.3% to the Latin American and Caribbean Group (GRULAC) and 2.8% to the African Group. The median time of completion for the first survey was 15 min, while the second had a median of 8.5 min (the assessment of faces was not included in this version, hence the lower duration). All participants self-reported being proficient in English.

The mean scores from the evaluation of the accented voices are shown in Table 1, except for the perception of gender, since all voices were perceived as belonging to male gender, with voting percentages close or equal to a 100%. Most of the voices were voted to be representative of a nationality clearly related to the intended accent or with a sensible second most voted option, e.g., Iran for *Arabic*_*G* *s*_ or Nigeria for *Kenya*_*M*_. The voice *Mandarin*_*O*_ stands out as having a particularly surprising most voted option. The results further suggest that accents from countries in the Global North are easier to determine by participants, even for simulated accents. The only exceptions are the Hindi and Singapore^16^ accented voices, which, respectively were and were not easily recognised by the participants.

**FIGURE 3:**
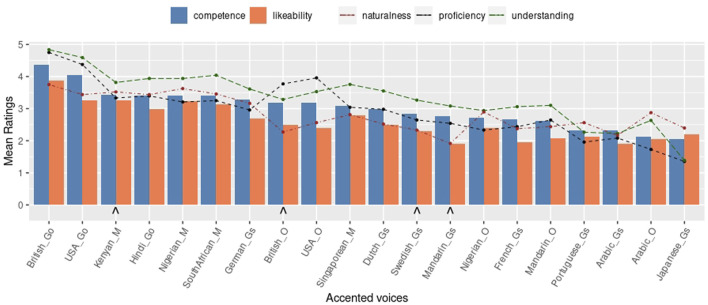
Online survey mean scores of easiness of Understanding, Naturalness, perceived Competence, Likeability and perceived English Proficiency. ∧ maker indicates voices selected for the robot study.

The official accented English voices from Google TTS, i.e., *British*_*Go*_, *American*_*Go*_, and *Hindi*_*Go*_, received the highest values for most of the measurements, while, the official set from Microsoft Azure’s TTS, i.e., *Kenya*_*M*_, *Nigeria*_*M*_, *Singapore*_*M*_ and, *SouthAfrica*_*M*_, received slightly lower ratings. Of the simulated accents, the *German*_*G* *s*_ voice received the highest ratings, while the others—such as *Dutch*_*Gs*_, *Swedish*_*Gs*_, *Mandarin*_*Gs*_, *Japanese*_*Gs*_ or *Arabic*_*Gs*_—had predominantly low ratings. In particular, the unexpectedly low rating for likeability for the *French*_*Gs*_ voice can be noted. The voices trained with the OverFlow technique received varied ratings, with the two native accents, *British*_*O*_ and *American*_*O*_ being rated higher than the others. Figure 3, which displays the accents in descending order of perceived competence, shows that the official commercial accents, i.e., Google and Microsoft Azure, were perceived as most competent (except for *Singapore*_*M*_), followed generally by western Europe accents (*French*_*Gs*_ and *Portuguese*_*Gs*_ being the exceptions) and Arabic accented English rated low regardless of synthesis option. Figure 3 further shows that perceived competence is strongly correlated with likeability, *ρ* = 0.89 (*p* = 1.12*e* ^−7^), perceived English proficiency *ρ* = 0.95 (*p* = 2.17*e* ^−10^), and with easiness of understanding *ρ* = 0.95 (*p* = 2.46*e* ^−10^). The correlation between perceived competence and naturalness is moderate, but also significant, *ρ* = 0.72 (*p* = 3.35*e* ^−4^).

The evaluation of the seven rendered faces, shown in Table 2, indicates consistent low assessment of perceived competence, naturalness and likeability. No statistical difference is found between any of the pair-wise comparisons. It should be noted, however, that the rating was based on a still picture only, and that the information provided to the participants was hence much more restricted than for the voices. The votes assigning a nationality to each rendered face were also diverse, with most faces nevertheless being perceived as belonging to a geographic region (face *A* to the Middle-East, *B* and *E* to Europe, *C* to East-Asia, *D* and *F* to Equatorial Africa), with only face *G* receiving conflicting nationality labels. Perceived gender was also inconclusive for most faces, displaying a clear consensus for face *A*, and lower majorities for faces *B*, *E* and *G*.

**TABLE 2:** Results of the evaluation of the face renders on (top 2) perceived Nationalities and Gender, and mean scores for perceived Competence, Naturalness and Likeability. Highest mean score per category indicated in bold font, lowest in italics. → indicates faces selected for the robot interaction study.

Face	Nationality (%)	Competent	Natural	Likeable	Gender (%)
A	Iran (39), Syria (21)	2.62	2.42	2.00	Male (100)
→ B	Poland (29), Sweden (19)	2.53	2.38	1.98	Male (70), Female (15), Non-Binary (15)
→ C	China (35), Korea (21)	*2.38*	*2.06*	*1.78*	Female (63), Male (21)
→ D	Kenya (44), Nigeria (42)	2.62	2.38	2.18	Male (44), Female (42)
E	Germany (29), France (17)	2.80	2.40	2.22	Female (85), Non-Binary (8)
F	Nigeria (44), Kenya (38)	2.67	2.29	**2.27**	Male (46), Non-Binary (15)
→ G	Iran (29), France (15)	**2.93**	**2.49**	2.20	Female (79), Non-Binary (10)

#### 9.1.1 Selection of an appearance and accent for robot interaction

Taking into account the small differences in the ratings of the face appearances, we used the scores assigned to the accented voices as a primary factor to select the representations that are to be used in the physical robot interaction. After following the procedure detailed in Section 7.2, the *British*_*O*_, *Mandarin*_*Gs*_, *Swedish*_*Gs*_ and *Kenya*_*M*_ accented voices were deemed to be sufficiently understandable, comparable in naturalness, and placed in opposite ends of perceived competence and likeability (*British*_*O*_, and *Kenya*_*M*_ being rated higher than *Mandarin*_*Gs*_ and *Swedish*_*Gs*_). The *Mandarin*_*Gs*_ voice was combined with face *C* and the *Kenya*_*M*_ voice with face *D*, as both pairs matched on perceived nationality, as shown in Tables 1, 2. The *Swedish*_*Gs*_ voice was paired with face *B*, which was perceived to be of Swedish background after the Polish option. Finally, the *British*_*O*_ voice was combined with face *G*, as only two rendered faces, *F* and *G*, received votes indicating a perceived British nationality and face *G* was more dissimilar to the faces used for the other options.

Furthermore, the combination of voice and face process considered pairs that received contrasting ratings regarding their combined perceived competence. The “British” face and voice combination was perceived as more competent (2.93 + 3.19 = 6.12) than the “Chinese-Mandarin” and “Swedish” combinations (2.38 + 2.77 = 5.15 and 2.53 + 2.83 = 5.36, respectively). The difference between the “British” and the “Chinese-Mandarin” robot is statistically significant (*p* = 4.36*e* ^−5^), as well as that between the “British” and the “Swedish” robot (*p* = 1.09*e* ^−3^). The “Kenyan” combination was also perceived as significantly more competent (2.62 + 3.44 = 6.06) than both the “Swedish” (*p* = 6.44*e* ^−4^) and “Chinese-Mandarin” (*p* = 1.8*e* ^−5^) combination. The differences between, respectively, the “Swedish” and the “Chinese-Mandarin”, and the “British” and “Kenyan” robots were non-significant.

### 9.2 Robot interaction

The robot interaction study involved 81 participants who were each presented with a single robot-nationality representation, following a between-subject design. The study was carried out in two separate instances, once in October 2022 with the British and Chinese robots and 28 participants, and one in April 2023 with the Kenyan and Swedish robots and 53 participants (a larger number of subjects was included in the second instance to be able to address the secondary research question of whether aligning the nationality-representation to one subject group had any effects). Since the set-up and procedure were identical between the instances and a between-subject design was used, we present the merged results. Data from 7 participants were excluded from the analysis due to partial loss of responses, technical problems, and failed data entry. In the cohort of remaining subjects, 37 participants self-reported as female, 35 as male, 1 transgender-female and 1 non-binary. The average age of the participants was 27.53 years (SD: 10.60). All participants were residents of Sweden, with nationalities including Sweden (31), India (6), France (4), China (3), Italy (3), Spain (3), Bangladesh (2), Germany (2), Hungary (2), Iran (2), Mexico (2), Ukraine (2), Australia, Belarus, Czech, Croatia, Greece, Ireland, Iraq, Kazakhstan, Serbia, Singapore, Switzerland and Vietnam. The distribution of international participants, when grouped into regional clusters, is similar to that obtained in Section 9.1, with 37.2% participants’ nationalities belonging to WEOG, 18.6% to Eastern European Group, 39.5% Asia and the Pacific Group and 4.7% to GRULAC. All participants self-reported as fluent in English, but this skill was not tested during the study. Participants were randomly assigned to one of the four robot-nationality condition, controlling for an equal distribution of gender and of Swedish subjects. No participants, other than the Swedish participants, were exposed to robot representations that aligned with their own reported nationality. The robot presentation took an average of 6.65 min (SD: 0.71).

**FIGURE 4:**
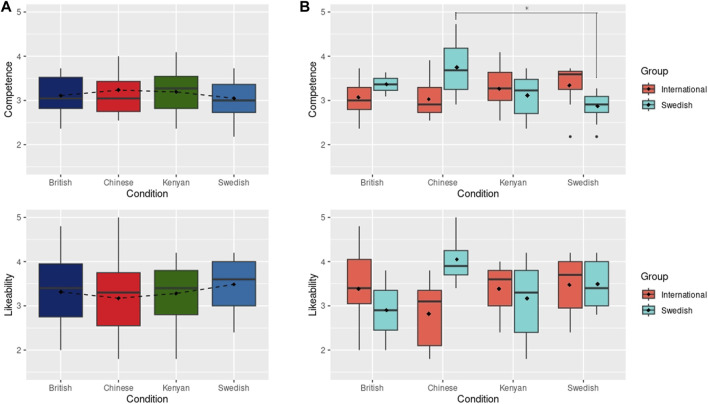
**(A)** Results of perceived Competence and Likeability after physical robot interaction. Dashed line connects the mean values (shown with black diamonds). **(B)** Results of perceived Competence and Likeability after the physical robot interactions grouped by International and Swedish participants. Black diamonds show mean scores. * indicates statistically significant difference at *p* < 0.05.

The ratings from the post-questionnaire are presented in Table 3 and Figure 4A. The nationality and gender labels have clear majorities for the Kenyan and Swedish robots, but not for the British and Chinese. For the British robot GB was the most frequently selected option (GB: 29%), but a similar number of votes were also obtained for Sweden (other answers include US, Brazil and Germany) and only half of the participants perceived the robot as being of male gender (note, however, that 72% of the respondents in the online survey perceived the face as being female). The results for the Chinese condition are more surprising, showing that Chinese nationality is selected only after *“None of the options*,*”* and followed by 7 other nationalities (including Korea, France, Great Britain, and Poland). The most voted gender was male (64%, which may be compared to 21% for the face-only picture in the online survey).

**TABLE 3:** Results for most voted perceived Nationality and Gender, mean easiness of Understanding, Naturalness, perceived English Proficiency, Human-likeness and Anthropomorphism. M, Male; F, Female; NB, Non-binary; GB, Great Britain. ** indicates statistically significant differences at *p* < 0.01.

Condition	Nationality (%)	Gender (%)	Understand	Natural	Proficient	Human	Anthro
British	GB (29), Sweden (21)	M (50), F (21)	1.86	2.29	3.14**	2.29	2.10
Chinese	None (29), China (21)	M (64), NB (21)	2.29	2.50	2.86	2.53	2.46
Kenyan	Nigeria (68), India (24)	M (92)	2.52	2.28	2.96**	2.47	2.58
Swedish	Sweden (81)	M (90)	2.43	2.24	2.00**	2.47	2.50

While the four tested robot portrayals appear to elicit a similar perception of human-likeness and anthropomorphism, it may first be noted that the mean values are rather low (slightly below or above the middle on the scale from 1 to 5). Similar values are found for naturalness, but in this case, the scores assigned to the robots contradict those of the online survey, in which the voices and faces were presented separately and the stimuli was much briefer (two synthesised sentences and a still picture). For example, the Chinese robot obtains a better score of 2.29 in naturalness, compared to the *Mandarin*_*Gs*_ voice and *C* face which scored 1.94 and 2.06, respectively. The Kenyan condition, instead, decreases from 3.55 to 2.38 assigned to the *Kenya*_*M*_ voice and face *D*, respectively, to a score of 2.28 for the robot. Furthermore, an ANOVA for the perceived proficiency indicated a significant difference between pair-wise comparisons. A Tukey-HSD test showed a significant difference between the Swedish-British conditions (*p* = 0.0059) and Swedish-Kenyan conditions (*p* = 0.0074).

Regarding the results for *perceived competence* (H1), an analysis of variance (ANOVA) did not reveal any statistically significant differences among the conditions, as depicted in Figure 4A. Notably, the mean rating of competence for the Chinese condition is considerably higher than that assigned to the *Mandarin*_*Gs*_ voice, indicating a more positive perception after interaction than after first impression. The three other conditions have similar values to those reported in the online survey. The results for *likeability* (H1) also show no statistical difference between conditions after an ANOVA test, although the Swedish robot obtained higher ratings that the other three robots (bottom of Figure 4A).

As discussed in Section 3, perceptions of robots and social agents, in voice and appearance, may differ even when people perceive the agent as aligned or not aligned with their own nationality. We hence extend our analysis to evaluate this phenomenon. When the participants are grouped by the sub-groups of nationality, i.e., Swedish (42%) and international (58%), the results differ from the overall rating. The *international* participants rate the Swedish robot with the higher *partner competence* scores (M: 3.34, SD: 0.541), then the Kenyan versions of the robot (M: 3.27, SD: 0.473), and the British (M: 3.07, SD: 0.434) and Chinese (M: 3.03, SD: 0.433) robots with lower values, as shown in the top of Figure 4B. These differences, however, are not significant. On the other hand, the *Swedish* participants rate the Swedish robot with much lower *partner competence* scores (M: 2.87, SD: 0.316) than the British (M: 3.36, SD: 0.386), Chinese (M: 3.75, SD: 0.790) and Kenyan (M: 3.11, SD: 0.465). An ANOVA test indicates a significant difference between these conditions. A following Tukey-HSD test shows a significant difference between the ratings assigned by Swedish participants to the Swedish and Chinese robot conditions on *partner competence* (*p* = 0.011). Notably, the Swedish participants rated the Chinese-encoded robot as most competent and likable compared to any other nationality representation.

The results for likeability show a similar trend with the *international* participants giving the *Swedish* robot the highest mean score (M: 3.48, SD: 0.658), followed by the British (M: 3.38, SD: 0.867) and Kenyan (M: 3.38, SD: 0.500) robots, and the Chinese robot (M: 2.82, SD: 0.757) receiving lower values than the rest, as shown in the bottom of Figure 4B. The *Swedish* participants, on the other hand, find the Chinese robot more likeable (M: 4.05, SD: 0.681) than they find the Swedish (M: 3.49, SD: 0.587) and Kenyan (M: 3.17, SD: 0.785) robots. The British robot is rated with the lowest values (M: 2.9, SD: 1.27). These differences are not significant.

**FIGURE 5:**
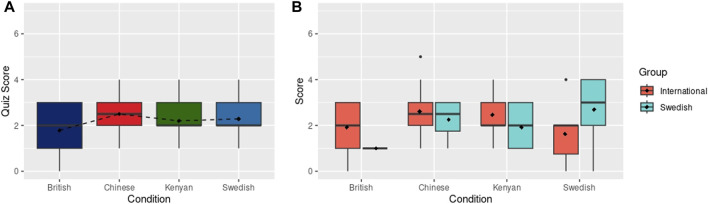
**(A)** Recall quiz scores after physical robot interaction. Dashed line connects the mean scores (shown with black diamonds). **(B)** Recall quiz scores after the physical robot interaction grouped by International and Swedish participants. Black diamonds show mean scores.

Finally, the quiz scores, used to measure recall of the information shared during the presentation, had no statistically significant differences between conditions, as shown in Figure 5A. However, the mean quiz score for the British condition was lower (M: 1.79, SD: 1.12) than the average score for the Swedish condition (M: 2.29, SD: 1.35), with the Kenyan (M: 2.20, SD: 0.866) and Chinese (M: 2.5, SD: 1.16) robots resulting in mean quiz scores in between. These results do not show support for H2 (better recall of information with robot that scored higher values in perceived competence and likeability), as the expected nationality-encoded robots with higher perception scores in the online survey, Kenyan-followed by the British-encoded robots, did not produced better quiz scores. When focusing on the sub-group of Swedish subjects, some non-significant differences are found as shown in Figure 5B. The *Swedish* participants perform considerably better (M: 2.69, SD: 1.25) than the *International* participants (M: 1.62, SD: 1.30) with the Swedish robot, whereas the *International* sub-group obtained better scores than the *Swedish* sub-group for the rest of robot conditions. However, these differences are not significant.

Regarding H3 (preference and better recall when a participant’s own identity aligns with a robot), the *Swedish* subjects rated the Swedish robot as being less competent and somewhat likeable, only liking the Chinese robot better than the Swedish, which does not provide complete support for H3 (Figure 4B).At the same time, however, *Swedish* participants scored, non-significantly, higher on the quiz with the Swedish robot. There is hence some, inconclusive, evidence that the Swedish subjects were more negative towards the Swedish robot (comparable to the findings by, e.g., Obremski et al., 2022), but despite this recalled more of what the robot had been telling them.

## 10 Virtual robot study

The difference between the results obtained from the online survey and the robot interaction may suggest that participants established different mental models of the robot in the two experiments. Since participants of the online survey had shorter time exposure to the voice/appearance of the virtual robot, compared to those interacting with a physical robot, it is likely that the former evaluations corresponded to immediate, possibly unreliable, first impressions (Cafaro et al., 2012). Research in agent and robot interaction has demonstrated that first impressions are not consistent and could be altered with longer interactive exposure (Paetzel and Castellano, 2019; Paetzel et al., 2020; Paetzel-Prüsmann et al., 2021). However, an additional difference exists in the online survey introducing a virtual robot and robot study having a physical. Therefore, an interactive study with a virtual robot was implemented to assess if the participants’ perception of the robot after this interaction would correlate with the results of the online survey (i.e., first impression) and/or the robot study (i.e., longer physical interaction). This study used the method described in Section 8 for the robot interaction, but interchanged the Furhat robot with its virtual version and performed the art presentation though a video-call platform. Only the Kenyan and Swedish versions of the robot were used in this study, in order to be able to investigate H3 (nationality-alignment) with a lower total number of participants (as the Swedish participants were spread over two, rather than four conditions). The Kenyan version was selected as it had the most similar ratings of easiness of understanding, naturalness, proficiency, human-likeness and anthropomorphism in the physical robot interaction study. The presentation, robot behavior, measurements and participant recruitment criteria were not modified.

### 10.1 Results

The virtual robot interaction was presented to 32 participants recruited through Prolific and the university campus. The mean age of the participants was 29.0 (SD: 7.21). Participants were residents of Sweden and included the following background nationalities: Sweden (17), Poland (2), Finland (2), Spain, Norway, Nigeria, Colombia, Netherlands, Italy, Indian, United States, Canada/Sweden, Sweden/Iraq, and Russia/United Kingdom. The distribution of nationalities among international participants, when grouped across regions, resembles the one shown in Section 9.2, with 66.7% participants belonging to WEOG, 6.7% to Eastern European Group, 13.3% Asia and the Pacific Group, 6.7% to GRULAC and 6.7% to the African Group. When prompted for gender, 19 participants self-reported as female and 13 as male. The distribution of gender and nationality per condition was comparable.

Table 4 shows the results for perceived robot nationality and gender, and mean ratings for easiness of understanding, perceived English proficiency, human-likeness and anthropomorphism. There is a clear majority for male gender for both robots, whereas the Swedish robot was labeled as being Swedish by a majority, whereas the Kenyan robot only reached 50% for the two Equatorial African alternatives Nigeria and Kenya, with the most frequent other options being India (12.5%) and None (12.5%). There is a significant difference in the perceived proficiency between the Kenyan (M: 2.88, SD: 0.719) and Swedish (M: 2.25, SD: 0.775) conditions (*p* = 0.024). This pattern is consistent with the results from the physical robot study. The remaining measurements in Table 4 have higher values for the Swedish condition, except for naturalness, but these differences are not significant.

**TABLE 4:** Virtual robot interaction results for most voted perceived Nationality and Gender, mean easiness of Understanding, perceived English Proficiency, Human-likeness and Anthropomorphism. * indicates statistically significant difference at *p* < 0.05.

Condition	Nationality %	Gender %	Understand	Natural	Proficient	Human	Anthro
Kenyan	Nigeria (31), Kenya (19)	Male (88)	2.63	2.19	2.88*	2.05	1.76
Swedish	Sweden (69), None (25)	Male (81)	3.13	1.94	2.25*	2.26	2.00

**FIGURE 6:**
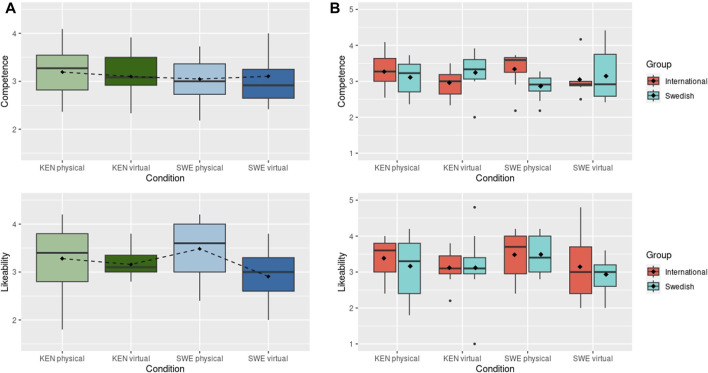
**(A)** Results of perceived Competence and Likeability with the Kenyan (KEN) and Swedish (SWE) conditions in the virtual and physical robot interaction. **(B)** Results of perceived Competence and Likeability grouped by International and Swedish participants. Black Diamonds show mean scores.

As shown at the top of Figure 6A there was no difference between the mean scores for *partner competence* for the Swedish (M: 3.104, SD: 0.625) and Kenyan (M: 3.098, SD: 0.516) virtual robots. There is also no statistical difference for the measurements of *likeability* for the Kenyan (M: 3.12, SD:0.816) and Swedish (M: 3.02, SD: 0.741) conditions, as shown at the bottom of Figure 6A. When compared to the physical robot condition, the results for *partner competence* show minimal variation from the virtual setting. On the other hand, the results for *likeability* of the Swedish condition diverge from those obtained with the physical robot. The Swedish physical robot was scored with higher values than the virtual robot, although non-significantly higher. Further, when comparing these values to those in the online survey for *perceived competence* (“Kenyan”: 2.62 + 3.44 = 6.06 and “Swedish”: 2.53 + 2.83 = 5.36) the trend for higher values for the Swedish condition remains in both virtual and physical robot settings. This trend is similar for the results of likeability in the online survey (“Kenyan”: 2.18 + 3.25 = 5.43 and “Swedish”: 2.00 + 2.29 = 4.29) and virtual robot, however, the results of *likeability* for the Swedish physical robot, on the other hand, are considerably higher than the Kenyan condition. These results may support the assumption that participant’s assessment in the online survey correspond to a first impression that is not representative of that after longer (interactive) exposure.

We next investigate if differences may be found between the Swedish (59%) and international (41%) participant groups. As shown in Figure 6B, the *Swedish* score more positively the *perceived competence* in both virtual robot conditions. This trend is reversed from the physical robot study, where the *International* participants give sightlier higher ratings to the physical robot conditions. These difference are less noticeable with the values for *likeability* for the virtual robots, also confirming that both groups rated the Swedish physical robot with higher values than the virtual robot.

**FIGURE 7:**
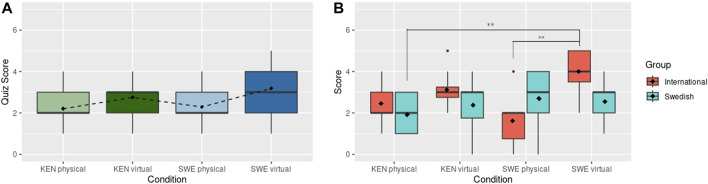
**(A)** Recall quiz results after the virtual and physical robot interaction. Dashed line connects the mean scores (shown with black diamonds). **(B)** Recall quiz results grouped by International and Swedish participants. Black Diamonds show mean scores. ** indicates statistically significant differences at *p* < 0.01.

The difference between the quiz scores for the Kenyan nationality condition (M: 2.75,SD: 1.18) and Swedish condition (M: 3.19, SD: 1.22) was not significant, but reversed the direction of the perceptual ratings, as the Kenyan robot produced lower scores than the Swedish robot, as shown in Figure 7A compared to Figure 6A). There is also a statistical difference between the Sweden virtual condition compared to the Kenyan physical condition (*p* = 0.0435). When we look at participants’ nationality, as shown in Figure 7B, it is notable that the *International* participants interacting with the Swedish virtual robot have a significantly higher score compared to the *Swedish* participants in the Kenyan physical robot (*p* = 0.0026) and compared to the *International* participants in the Swedish physical robot condition (*p* = 0.0013). Both differences are denoted in Figure 7B with asterisks. More importantly the *International* participants scored considerably better than the *Swedish* participants in the same condition, i.e., Swedish virtual robot. This result reverses the effect seen with the Swedish physical robot.

## 11 Discussion

In this paper, our objective was to investigate how the portrayal of nationality through the voice and appearance of a virtual and physical social robot could elicit perceptions related to stereotypes. The results of the online survey suggest that these preconceptions are present in the evaluation of the accented voices. The perceived competence (and the correlated measurements for likeability and perceived English proficiency) assigned to non-western accented voices were mostly rated lower than the western counterparts, even when comparing voices rated as equally intelligible. The voices furthermore received mostly clear majority votes for a perceived nationality and gender. On the other hand, for the picture of the virtual robot’s face appearance there were no significant differences in perceived competence, nor were they as clearly associated with a perceived nationality or gender, and the rendered faces moreover received consistent low ratings, in contrast to the greater range of scores assigned to the accented voices. These results seem to follow previous findings that accents dominate over looks for perception of ethnic or national category (Krenn et al., 2017).

The main study explored if the same subject perceptions would be found for after an interaction with a social robot. However, the results did not reveal any difference when evaluating robots depicting a British, Chinese, Kenyan or Swedish nationality. Consequently, H1, which suggested that the ratings for competence and likeability in the online survey would carry over to the physical robot condition, did not find support in this study. One explanation for this may be that the de-contextualized stimuli presented in the online survey—short audio clips of the accented synthetic speech and faces displayed as images only—may have prompted responses to stereotypes from the participants, whereas the nationality factors were less important to them in the actual interaction with a robot. Note that this appears to differ from studies in human-human interaction that indicate that first impressions are often maintained even after longer interactions. However, this effect may be reversed when it comes to social robots (Paetzel and Castellano, 2019; Paetzel et al., 2020). A related interpretation could be that the evaluation of individual components of a social robot, i.e., voice and appearance separately such as in the online survey, makes differences in these components stand out and trigger stereotypical perceptions, but when combined together with other elements in the interaction, the perception of these individual factors is overshadowed by the overall experience of the interaction and the ratings instead reflect their overall assessment of the interaction to different questionnaire questions. The open comments received after the study support this and points to a general problem when evaluating specific factors of a robot’s properties. The comments indicate that participants had a high expectation of the interactive abilities of the robot, and if their expectations were not met regarding general TTS quality, responsiveness of the robot in the interaction or the content of the presentation, they may have become less responsive to the manipulated variables of the study. Previous research has shown that conversational robots are harshly criticized after they made a mistake in comparison to robots that only use functional speaking (Cha et al., 2015) and that robots taking less rigorous roles, e.g., as a peer, are allowed to make mistakes (Tanaka and Matsuzoe, 2012). The setting and role of the robot may hence have influenced the results, since a robot art presenter that is knowledgeable regarding the facts about the artist may be expected to also be linguistically proficient and easy to understand. The participants therefore likely penalised the robot versions mispronouncing names and years.

Regarding H2, the results did not provide evidence to support the hypothesis that participants would exhibit improved information recall when interacting with a physical and virtual robot that received higher ratings in terms of perceived competence and likeability in the online survey. In fact, the quiz results consistently displayed lower values across most study conditions, with the exception of the Swedish virtual robot condition, particularly when interacting with the international participant group. A partial explanation for this discrepancy is that participants in the online setting were encouraged to use a headset and a laptop/computer during the experiment that might have contributed to better overall recall. This reason might lead to the better quiz scores linked to the virtual settings. However, since the International group shows a significantly higher performance, a more compelling reason might be rooted in the fact that international participants are likely to have more exposure to a Swedish accent in English, as speaking English with Swedish colleagues is more likely among international individuals. On the other hand, Swedish participants may have found the simulated accent to be more surprising or unexpected. Following the findings of Van Engen and Peelle (2014), the familiarity to a Swedish accent in English may have reduced the cognitive load of the International group producing better results.

For hypothesis H3, the effects of culturally aligning the robot to the user group, the results for the Swedish robot, as well as the results from the sub-groups of participants, are intriguing. In the physical and virtual robot study, the Swedish participants rate the Swedish robot with the lowest partner competence score compared to other robot nationalities. However, Swedish participants were not so extreme when rating the likeability of the Swedish robot, as it received medium scores, with other nationality-encoded robot obtaining greater or lower mean scores. Notably, Swedish participants awarded significantly higher scores for perceived competence and likeability to the Chinese physical robot when compared to other robots. The results from the recall quiz further highlight disparities, with better performance observed with the Swedish physical robot among Swedish participants, while in the virtual robot condition, International participants achieved significantly higher mean recall scores. Here, it is important to reiterate the before mentioned point. While all participants enrolled in this study were residents of Sweden, it is likely that the reaction to the unnaturalness of the *Swedish*_*Gs*_ voice (2.32 out of 5) was perceived as more critical for the Swedish participants. Given the stronger influence of accents on perception of individuals’ cultural categorization (Rakić et al., 2011), this may have prompted a more critical evaluation of the robot in terms of competence. As described in section 3.1, negative impressions when there is alignment to one’s accent (Niculescu et al., 2008; Batsaikhan et al., 2021; Obremski et al., 2022) or dialect (Torre and Maguer, 2020) have been reported in previous work.

Furthermore, when we looked closer to the values reported for mean easiness of understanding, we see a slight correlation with the results of the recall quiz, i.e., British lowest and Swedish second highest. However, the values assigned for perceived speaking proficiency show a different pattern, i.e., British highest and Swedish lowest, and it is hence clear that the participants regard easiness of understanding and proficiency as separate dimensions. This is, however, in line with findings for natural human accented speech (Tergujeff, 2021).

### 11.1 Limitations

We attempted to gather different accented voices in English from commercially available TTS services as well as open datasets to train our own synthesizers, but the options were highly restricted. The range of voices that we could employ in the study was thus limited, as were the factors that we could control in the synthesized voices, e.g., loudness, pitch, and extroversion, which have been shown to affect the users’ ratings of interactions (Niculescu et al., 2008). Additionally, the content, both lexical or gestural, generated during the interaction was not tailored to any of the nationality-encoded representations. While this approach enables an accurate study of the influence of individual factors, i.e., accent and face appearance in this study, it is worth noting that an individual’s lexicon and behavior are intricately linked to their background, which poses a considerable limitation on how our robots’ behaviors were designed.

## 12 Conclusion

We have reported on three studies focused on exploring how stereotypes may transfer to human-robot interaction. The first, the online survey, was employed to validate voice and face representations of nationality in a virtual or physical robot, and corroborate that these representations received ratings and nationality labels associated with stereotypes. The results are in line with previous work on human-human interaction, since they indicate that, in particular, accents lead to preconceptions on perceived competence and likeability that align with negative and positive stereotypes. The main study, which followed up on this, explored if these stereotypical perceptions were the same after actual interaction with a social robot. For this study, the results do not correspond to expectations from human-human interactions that have shown that stereotypical positive bias towards Western countries is maintained over time and longer interactions. The robot using a Mandarin-accented voice and a stereotypical Asian face and a Kenyan-accented voice with a stereotypical African face were rated at least as favourably (no statistical differences) in terms of perceived competence and likeability as a robot with a British- or Swedish-accented voice combined with faces perceived as being European. Future work is thus needed to firstly explore more human-robot interaction settings to see if the findings are valid in other HRI scenarios and secondly corroborate if the results hold also when improvements to the TTS permits to avoid technology-induced pronunciation mistakes. A final, very important, and fundamentally positive note is nevertheless that the study shows that prejudice regarding different nationality stereotypes does not seem to be a strong factor in human-robot interaction—at least not for the included set of subjects. The present subject group of young, university students in an international setting have traits (age, educational level and exposure to different cultures, multilingual competence) that have been shown to lead to more cultural open-mindedness and higher acceptance of, e.g., accented speech (Boduch-Grabka and Lev-Ari, 2021; Dekker et al., 2021).

## Data Availability

The raw data supporting the conclusion of this article will be made available by the authors, without undue reservation.

## Footnotes

1
https://learn.microsoft.com/en-us/azure/ai-services/speech-service/language-support

2
https://docs.aws.amazon.com/polly/latest/dg/voicelist.html

3
https://www.acapela-group.com/voices/available-languages/

4
https://furhatrobotics.com/

5
https://www.hansonrobotics.com/sophia/

6
https://www.engineeredarts.co.uk/robot/ameca/

7
http://www.geminoid.jp/en/robots.html

8 The notion of different human races is rejected by many international bodies, such as the UN general assembly and the European Union, but since this is the term used by Bartneck et al. (2018) and Sparrow (2020), we reuse the term when describing these arguments. In the rest of the paper, the use of this term is only done when the original authors employ it.

9 The use of these terms is attributed to the original work by Sparrow (2020) and do not reflect the terminology accepted in this work.

10 This term is used by Makatchev et al. (2013) in the original work and diverges from our interpretation of nationality.

11
https://cloud.google.com/text-to-speech

12
https://azure.microsoft.com/en-us/products/cognitive-services/text-to-speech/

13 “When the sunlight strikes raindrops in the air they act as a prism and form a rainbow. The rainbow is a division of white light into many beautiful colors.”

14
https://en.wikipedia.org/wiki/Dorothea_Tanning

15
https://www.un.org/dgacm/en/content/regional-groups

16 While the list of countries included in the Global North can change with different contexts, Singapore is often considered part of this classification.
